# Can cryptic female choice prevent invasive hybridization in external fertilizing fish?

**DOI:** 10.1111/eva.13573

**Published:** 2023-07-13

**Authors:** Tyler H. Lantiegne, Craig F. Purchase

**Affiliations:** ^1^ Department of Biology Memorial University of Newfoundland St. John's Newfoundland & Labrador Canada

**Keywords:** alternative reproductive tactics, conspecific sperm preference, mate choice, polyandry, post‐copulatory sexual selection, *Salmo salar*, *Salmo trutta*, *Salvelinus fontinalis*, sneaker males, sperm competition

## Abstract

Polyandrous mating systems result in females mating with multiple males, generating opportunities for strong pre‐mating and post‐mating sexual selection. Polyandry also creates the potential for unintended matings and subsequent sperm competition with hybridizing species. Cryptic female choice allows females to bias paternity towards preferred males under sperm competition and may include conspecific sperm preference when under hybridization risk. The potential for hybridization becomes particularly important in context of invasive species that can novelly hybridize with natives, and by definition, have evolved allopatrically. We provide the first examination of conspecific sperm preference in a system of three species with the potential to hybridize: North American native Atlantic salmon (*Salmo salar*) and brook char (*Salvelinus fontinalis*), and invasive brown trout (*Salmo trutta*) from Europe. Using naturalized populations on the island of Newfoundland, we measured changes in sperm swimming performance, a known predictor of paternity, to determine the degree of modification in sperm swimming to female cues related to conspecific sperm preference. Compared to water alone, female ovarian fluid in general had a pronounced effect and changed sperm motility (by a mean of 53%) and swimming velocity (mean 30%), but not linearity (mean 6%). However, patterns in the degree of modification suggest there is no conspecific sperm preference in the North American populations. Furthermore, female cues from both native species tended to boost the sperm of invasive males more than their own. We conclude that cryptic female choice via ovarian fluid mediated sperm swimming modification is too weak in this system to prevent invasive hybridization and is likely insufficient to promote or maintain reproductive isolation between the native North American species.

## 1 INTRODUCTION

Sexual selection can occur via intrasex competition between individuals for access to mates and fertilizations, and intersex via mate choice for the opposite sex (Jones & Ratterman, 2009; Kuijper et al., 2012). Females are typically the choosier sex and select mates based on various attributes, including body odor (Ferkin, 2018) and courtship displays (Jennions & Petrie, 1997). Males, therefore, usually invest a large amount of energy into creating mating opportunities, while females invest comparatively more energy in the production of gametes and parental care (Bateman, 1948; Emery Thompson & Georgiev, 2014; Trivers, 1972). This difference of energetic expenditure between males and females creates situations where females may benefit (Firman, 2011) from mating polyandrously, with more than one male, to better increase her chances of mating with high‐quality males and producing good quality offspring.

In polyandrous mating systems (Kekäläinen & Evans, 2018; Pizzari & Wedell, 2013), a female's eggs are exposed to sperm from many males, potentially creating the context for sperm competition to occur (Parker, 1970). In some situations, polyandry can result in fertilization by males of a different species, which facilitates hybridization (Garner & Neff, 2013; McGowan & Davidson, 1992; Tynkkynen et al., 2009). Across taxa, hybrid matings can result in highly variable outcomes, including speciation (Abbott et al., 2013), fertile or sterile hybrid offspring (Close & Bell, 1997), or no offspring due to failed fertilization, abortion, or abnormal development (Buss & Wright, 1958; Chevassus, 1979; Wilson et al., 1974). The potential for inviable or sterile offspring creates energetic waste (Remick, 1992); females have more to lose than males with each hybrid mating and thus should avoid hybrid fertilizations.

Under post‐ejaculatory pre‐zygotic sexual selection, females can bias sperm competition towards preferred males via cryptic female choice (Eberhard, 1996; Firman et al., 2017; Thornhill & Alcock, 1983). The magnitude of this alteration can vary between males based on male relatedness to the female (Landry et al., 2001; Yeates et al., 2009), perceived social status (Firman et al., 2017) and quality (Dean et al., 2011). Mechanisms of cryptic female choice in internal fertilizers include manipulating the duration of copulation, favouring males that provide greater stimulation during copulation, transferring favored sperm to better locations within the reproductive tract, discarding unwanted sperm, removing copulatory plugs, and changing internal conditions to be more or less favorable for sperm (Dixson, 2003; Eberhard, 2010; Pizzari & Birkhead, 2000). External fertilizers do not have this degree of control. Therefore, hybridization is more difficult to avoid in external fertilizers when unchosen males release sperm simultaneously with the female's preferred mate.

However, externally fertilizing females can alter sperm behaviour using chemicals released with eggs, e.g., in mussels (Lymbery et al., 2017) and fish (Alonzo et al., 2016; Elofsson et al., 2006; Zadmajid et al., 2019). Generally, these chemicals improve sperm swimming performance compared to a water‐only environment (Elofsson et al., 2006; Lahnsteiner, 2002; Purchase & Rooke, 2020) and due to differential degree in response among males, subsequently bias fertilizations under sperm competition. Under hybrid matings, this form of cryptic female choice is known as conspecific sperm preference and allows a female to bias fertilization towards her own species (Castillo & Moyle, 2019; Yeates et al., 2013). When invasive species are introduced to a system, new opportunities for hybridization can occur (Biedrzycka et al., 2012; Muhlfeld et al., 2017), which by definition, is between individuals from allopatric populations. If pre‐mating mechanisms are inadequate, post‐mating pre‐zygotic conspecific sperm preference through cryptic female choice is the last line of defence to prevent hybrid fertilization (Birkhead & Pizzari, 2002; Yeates et al., 2013).

In studies using paired species, by definition there is one conspecific and one heterospecific species. Conspecific sperm preference changes conspecific sperm swimming performance differently than sperm from heterospecific males (Castillo & Moyle, 2019; Yeates et al., 2013). However, how females relatively bias sperm performance across several species of potential fathers has not been investigated. If there are multiple heterospecific species that can fertilize a female's eggs, they will not likely pose equal threats. How does the strength of cryptic female choice via conspecific sperm preference vary with multiple species? A good study system to examine this question is with external fertilizing salmonid fishes, as they are polyandrous (Haddeland et al., 2015; Lewis & Pitcher, 2017; Weir et al., 2010) and cryptic female choice mechanisms are reportedly strong (Butts et al., 2012; Rosengrave et al., 2016; Yeates et al., 2013) and readily manipulated. We chose three North American salmonids (see Table S1) that can produce hybrids (Chevassus, 1979); native brook char (*Salvelinus fontinalis*) and Atlantic salmon (*Salmo salar*), and brown trout (*Salmo trutta*), which were introduced from Europe and are considered one of the top 100 worst invasive species in the world (Lowe et al., 2000). Brown trout create hybrids with both Atlantic salmon (Chevassus, 1979) and brook char (Buss & Wright, 1958), while native brook char and Atlantic salmon rarely produce viable offspring as a product of natural mating (Chevassus, 1979)—although actual mating rates in the wild between these species are not known and could be high. If hybrid matings occur, hybrid fertilizations among all three species create evolutionary dead‐ends (Table S1; Blanc & Chevassus, 1979; Buss & Wright, 1958; Chevassus, 1979; Garcia‐Vazquez et al., 2004; Hartley, 1987; Lecaudey et al., 2018; Makhrov, 2008; McGowan & Davidson, 1992; Nygren et al., 1972; O'Connell, 1982; Sorensen et al., 1995; Sutterlin et al., 1977) and thus should be avoided by females if possible.

In their native Europe, salmon and trout are reported to show strong conspecific sperm preference that is mediated by ovarian fluid (Yeates et al., 2013), but North American populations of salmon and char have not been examined. Due to salmonid ovarian fluid creating an improved physical (Graziano et al., 2023) and chemical (Elofsson et al., 2006; Lahnsteiner, 2002; Lehnert et al., 2017) swimming environment for sperm compared to water, we hypothesized that (1) sperm respond positively to all ovarian fluid, when compared to swimming in only water, but (2) as a key mechanism to reduce the loss of eggs to hybridization, that ovarian fluid of all three species benefits the swimming of conspecific sperm more than that of heterospecific sperm. When there is more than one potential heterospecific species of father, we hypothesized (3) that there is a pattern in heterospecific modification that follows either (1) taxonomic relationships (e.g., distant related species have gametes that are not compatible and cannot fertilize each other, thus there is no need for conspecific sperm preference via changes to sperm swimming) or alternatively, the (2) likelihood of spawning interactions when gametes are compatible. For example, perhaps females of species A are more likely to be able to avoid hybrid fertilizations by males of species B than C, if A‐B normally spawn at the same time of year (could often be sperm competition) but A‐C do not (little chance of sperm competition). Consistency in heterospecific patterns across groups of species would inform on these alternative hypotheses.

## 2 METHODS

### 2.1 Experimental design

**FIGURE 1:**
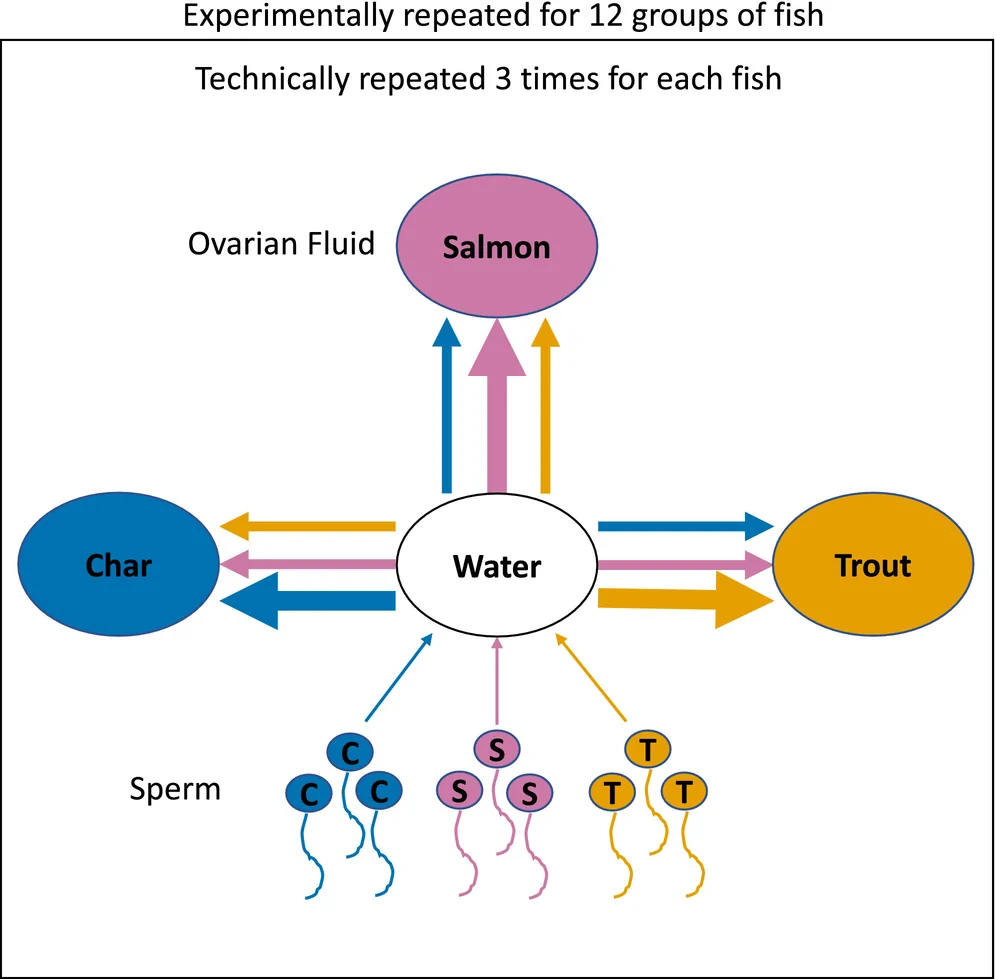
Schematic of the conceptual design. Small circles with tails are sperm, while large circles are ovarian fluid and water. Arrows represent sperm velocity in water or ovarian fluid (the same semen sample was tested in both as separate aliquots of individual sperm). Ovarian fluids were predicted to show conspecific sperm preference, indicated by greater modification of conspecific sperm (bolded arrows) over heterospecific sperm (un‐bolded arrows). Modification was quantified as the ratio of sperm swimming performance (from the same semen sample) in ovarian fluid compared to that in water, which controls for individual variation in male quality (variable performance among males in water). Experimental replication was achieved with 12 groups of fish, and sperm activations were technically repeated three times for each comparison.

In the presence of sperm competition in natural matings, females are exposed to sperm from multiple males, which creates opportunities to bias paternity. To examine the potential for cryptic female choice, using a split‐brood design, we took a sample of ovarian fluid from an individual female (diluted it), split it into three aliquots (~split‐brood), and exposed conspecific and two species of heterospecific sperm to it. We used a split‐ejaculate design (self‐controlled) to quantify the sperm swimming performance of individual males in ovarian fluids and a water standard (Figure 1) and then determined their ratio. This ratio allowed us to quantify modification with a standardized value that is independent of differences between the values (e.g., an increase of 30 units from 30 to 60, or 40 units from 40 to 80, gives the same ratio) and thus controls for confounding variables such as individual differences in male sperm quality (Gage et al., 2004; Purchase & Moreau, 2012). A key prediction is that each ovarian fluid species changes conspecific sperm more than heterospecific sperm (Figure 1), i.e., for ratios (ovarian fluid/water) all ovarian fluid species should create standardized values in the same direction for all sperm species (all >1, or for all <1), but the change should be greatest for conspecific sperm.

Sperm swimming performance comparisons were conducted over a series of experimental replicates (unique groups of fish), each containing one female and one male of each species. In each of these replicates, samples of ovarian fluid and sperm from each of our three study species were independently exposed to one another (different individual sperm, but importantly the same semen sample from each male was exposed to each ovarian fluid, in isolation from other males), Figure 1. Each sperm activation was technically repeated three times. Every experimental replicate tested each male in water, as well as individual samples of the three ovarian fluids (three males per replicate, each male's sperm activated 12 times, for a total of 36 sperm swimming comparisons per replicate). We ran 12 experimental replicates with different fish, with two replicates occurring on a given day. In two of the total 36 sampled males, preliminary assessment of semen quality was very poor, and we replaced that fish with the male from the other replicate on that day. Over the course of the study, we used 70 fish: 12 females of each species, and 12 brown trout, 11 brook char, and 11 Atlantic salmon males. To simplify analyses, we subsequently treated the two reused males as independent, as they were used with different females. We produced 432 sperm swimming comparisons (12 experimental replicates × 3 species of male × 4 sperm activation solutions × 3 technical replicates).

### 2.2 Fish collection

Population sizes of our study species were large enough not to be negatively affected by sampling. Fish were sourced from different places, but to avoid potential confounding variables, care was taken to ensure that all sperm could be examined at “exactly” the same amount of time from collection. As a species, salmon co‐evolved with trout and char, but North American salmon have been isolated from European salmon for 600,000 years (Lehnert et al., 2020), and are genetically different (e.g., Hartley, 1987). Brown trout co‐evolved with salmon in Europe but were not exposed to brook char, while our North American salmon co‐evolved with brook char. In judging whether or not cryptic female choice through conspecific sperm preference is an effective preventor of hybridization, it is important to note that in situations of novel invasion, populations are allopatric prior to contact between invasive and native species. Details of each sampled population are described below.

Wild native Atlantic salmon were sourced from the Exploits River in Newfoundland, Canada (48.93 N, 55.67 W). These co‐occur with native brook char in this watershed. Fish were trapped in the fishway on Grand Falls on September 7, 2018 and transferred to tanks on September 30, following previous protocols (Rooke et al., 2019). At ~11 AM on gamete collection days from November 2 to 14, individuals were anesthetized with MS‐222, paper toweled dry, and then stripped of gametes via ventral massage. Semen was stripped into plastic bags and eggs into glass jars.

Wild native brook char were collected from Star Lake in Newfoundland, Canada (48.58 N, 57.23 W). This is part of the Exploits River watershed, but there are no reports of salmon occurring within this particular lake. Char were captured via fyke net from September 21 to October 5, 2018, transported via truck, and housed in tanks at the same facility as the salmon. Brook char were fed a diet of mealworms until October 5 and then fed 4 mm biobrood pellets for the remaining duration of captivity (salmon do not eat before spawning and were thus not fed). Brook char were anesthetized with MS‐222. Females were stripped over the last week of October, the eggs were filtered out—see below, and ovarian fluid frozen. Freezing ovarian fluid does not change how it affects sperm swimming performance (Purchase & Rooke, 2020). Brook char males were stripped of semen immediately (minutes) after the salmon. Fresh char semen and frozen ovarian fluid were stored in 1.5 mL Eppendorf tubes. Gametes from salmon and char were transported on ice and received at the laboratory in St. John's at ~11 PM; all experimental procedures were done overnight and completed within 24 h of gamete collection.

Brown trout have not yet invaded the Exploits River watershed from which char and salmon were collected in central Newfoundland. Trout were introduced from Scotland in the late 19th century (Hustins, 2007) into watersheds surrounding St. John's and have since invaded throughout southeastern Newfoundland (MacDonald et al., 2022; Westley & Fleming, 2011). As invaders, these fish have been documented to hybridize with (McGowan & Davidson, 1992) and outcompete (Sorensen et al., 1995) native salmonids. Based on a generation time of 3–5 years, there were 27–35 generations of brown trout in Newfoundland at the time of collection. Wild, non‐native brown trout used in this study were captured via dipnet in tributaries of Windsor Lake (47.60 N, 52.78 W), in St. John's Newfoundland, where there are brook char but no Atlantic salmon. Trout were anesthetized immediately after capture, measured for length, fin‐clipped to avoid double sampling on different days, and stripped for gametes into plastic containers. Through coordinated field activities, trout stripping took place on the same days and at the same time (<1 h) as Atlantic salmon and brook char stripping. Trout gametes were kept on ice for ~12 h, the same duration as char and salmon before use. Animal care protocols did not allow the use of MS‐222 with fish (trout) being released back into the wild, and thus clove oil was used. Both anesthetics have been shown to have no significant effects to gametes when used prior to gamete collection (Holcomb et al., 2004).

### 2.3 Gamete preparation

An aliquot of semen (0.5 mL) from each male was centrifuged at 4100 *g* for 10 min at 5°C to separate seminal fluid from sperm. This seminal fluid acted as a non‐activating diluting agent to decrease the density of other aliquots from the same fish (Purchase & Moreau, 2012) at a 1:75 sperm to seminal fluid ratio. This minimized sperm clumping and allowed for high‐quality sperm data measurement. The ovarian fluid was filtered from eggs with a fine‐mesh aquarium net and refrigerated at 4°C in a glass beaker. Ovarian fluid activating solutions were made at 33% concentration with water. Bovine serum albumin was included in the sperm activating solution at a concentration of 1:1000 to prevent sperm from adhering to the microscope slide (Beirão et al., 2014, 2015).

Sperm swimming performance was recorded using a Prosilica GE680 camera attached to an inverted Leica DM IL LED microscope, with a 20× phase contrast objective. Approximately 1 μL of diluted semen was put on the edge of the chamber of a Cytonix 2 chambered slide, which had been cooled to ~9°C with a custom Physitemp TS‐4 system. The semen was then flushed into the chamber by 395 μL of the sperm activating solution (the test treatment). This activated the sperm and marked the start of the video, which was taken at 80 fps. The first 6 s post‐activation were used to locate an area of suitable sperm density (greater than 40 cells and less than 200; average of 88 cells were monitored at 6 s) and focus the microscope. Videos were captured using Streampix software, and quality checked for sperm density, motility, and proper microscope focus before being accepted into the data pool. If a video was deemed poor quality, the entire sperm activation process was repeated until three adequate videos were attained for technical replication (see above). Months later during computer analyses, 14 videos had to be removed from the pool after it was found that they did not have any sperm meeting definitions of swimming. Subsequently, we had data for 418/432 videos. We averaged the data among the “3” videos within a comparison for analyses, to simplify statistical analyses and account for the missing technical replicates in some cases (experimental sample size was not affected by having 2 vs. 3 technically repeated videos for a comparison).

### 2.4 Data analyses

Sperm swimming performance was determined from 6.0 to 20.0 s post‐activation, using the Computer Assisted Sperm Analysis (CASA) plugin in ImageJ with a tracking interval of 0.5 s (Purchase & Earle, 2012), Table S2. Decline of sperm swimming performance with time post‐activation in water is presented in Figure S1. Three sperm swimming performance traits were used in analyses (Alonzo et al., 2016; Evans et al., 2013; Gage et al., 2004; Lehnert et al., 2017; Young et al., 2013); the percent of the sperm cells within an ejaculate that are motile (MOT), and of the motile cells, their swimming linearity (LIN) and curvilinear swimming velocity (VCL). How ovarian fluid modified these parameters in different species of sperm – controlling for individual variation in sperm quality using a water standard, was our metric for determining conspecific sperm preference and thus the ability of females to exert cryptic female choice. To simplify analyses, we elected to focus this comparison to the earliest sperm post‐activation time period available (6.0–6.5 s) as fertilizations happen quickly (Beirão et al., 2019; Hoysak & Liley, 2001; Rosengrave et al., 2016), and thus represents the most important time interval for females to modify.

We used a mixed effected generalized linear model approach to test our hypotheses. Models were constructed using the lmer package. *p* values were generated using 2‐way ANOVAs. Assumptions of parametric statistics were tested by examining model residuals. To test hypothesis #1 that sperm swimming improves in ovarian fluid when compared to swimming in only water, we constructed mixed effects generalized linear models for motility (binomial error), LIN (normal error) and VCL (normal error), Equation 1. The binomial model was tested and not over dispersed. All three models used the fixed effect of sperm activating solution (water or ovarian fluid—the average of all types) and the random effect of male ID as the independent variables.

(1) $$\%\mathrm{mot},\mathrm{LIN},\mathrm{VCL}=\beta_{0}+\beta_{\text{Sperm Activating Solution}}+\beta_{\text{Male}\;\mathrm{ID}}+\varepsilon :\text{binomial},\varepsilon :\text{normal}$$

To evaluate hypothesis #2 that ovarian fluid changes conspecific sperm differently than heterospecific sperm we used two approaches. All three metrics (as standardized motility values were no longer proportions) were tested with normal distributed error. We created new linear models to test these hypotheses. First, we broke ovarian fluids into two categories for each male (conspecific [1 female] or heterospecific [average of 2 females]). We used standardized swimming performance (the ratio in ovarian fluid to water) as the dependent variable, ovarian fluid type (conspecific or heterospecific) as a fixed independent variable, and male ID as a random independent variable (Equation 2). A significant result from this model would indicate that across 36 females (ignoring their species) on average, modification of conspecific sperm would be different than the average of two species of heterospecific sperm.

(2) $$\%\mathrm{mot}\;\text{ratio},\mathrm{LIN}\;\text{ratio},\mathrm{VCL}\;\text{ratio}=\beta_{0}+\beta_{\text{Ovarian Fluid Type}}+\beta_{\text{Male}\;\mathrm{ID}}+\varepsilon :\text{normal}$$

Second, to conduct analyses at a finer resolution in case only some species of ovarian fluid support conspecific sperm preference, and to test hypothesis #3 that there are patterns of modification among the two heterospecific species of sperm within each ovarian fluid, we constructed new generalized mixed‐effects models and used standardized motility, linearity, and velocity as dependent variables, and sperm species, ovarian fluid species (char, salmon, or trout), and their interaction as fixed independent variables, and male and female ID as random independent variables (Equation 3). If the interaction was significant, the model was broken down by ovarian fluid species, and if necessary, analyzed post‐hoc with a Kenward‐Roger corrected Tukey test to determine differences among male species in each ovarian fluid.

(3) $$\begin{array}{c} \%\mathrm{mot}\;\text{ratio},\mathrm{LIN}\;\text{ratio},\mathrm{VCL}\;\text{ratio}=\beta_{0}+\beta_{\text{Sperm Species}}+\beta_{\text{Ovarian Fluid Species}}+\\ \beta_{\text{Sperm Species}\times \text{Ovarian Fluid Species}}+\beta_{\text{Male}\;\mathrm{ID}}+\beta_{\text{Female}\;\mathrm{ID}}+\varepsilon :\text{normal} \end{array}$$

## 3 RESULTS

**FIGURE 2:**
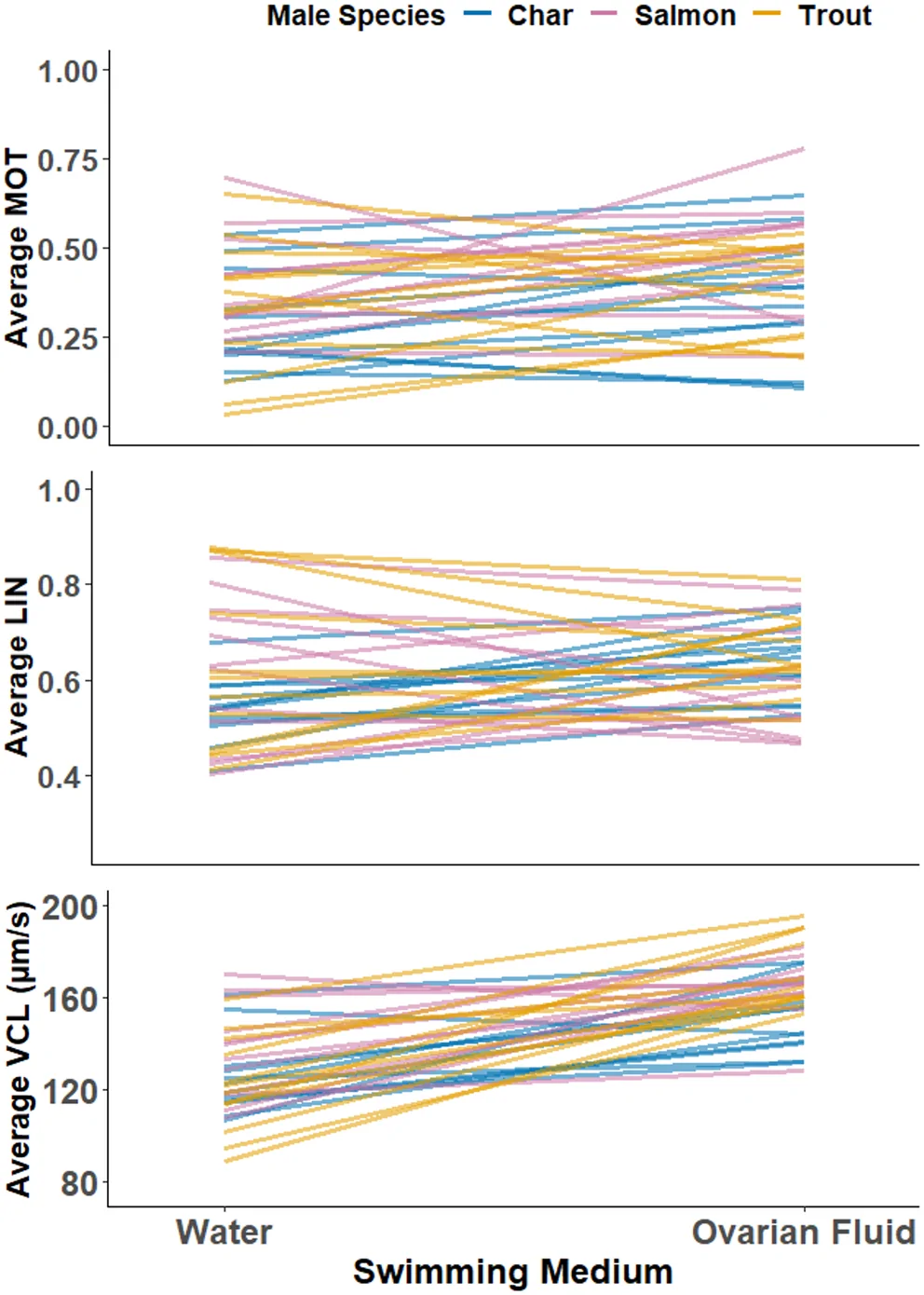
Reaction norms (top = proportion sperm motile, middle = swimming linearity, bottom = curvilinear swimming velocity) comparing sperm swimming performance from 6.0 to 6.5 s post‐activation in water to the average value in three ovarian fluid species. Each line represents an individual male (blue = char, pink = salmon, orange = trout) and is created by two points; means for water represent three technical replicate activations for each male, while those for ovarian fluid are from nine activations (three technical replications from each of three species of ovarian fluid). Positive slopes indicate up‐regulation of sperm swimming by ovarian fluid. Standardized (ovarian fluid/water) ratios >1.0 indicate positive up‐regulation and were on average 1.53 for MOT, 1.06 for LIN, and 1.30 for VCL.

**FIGURE 3:**
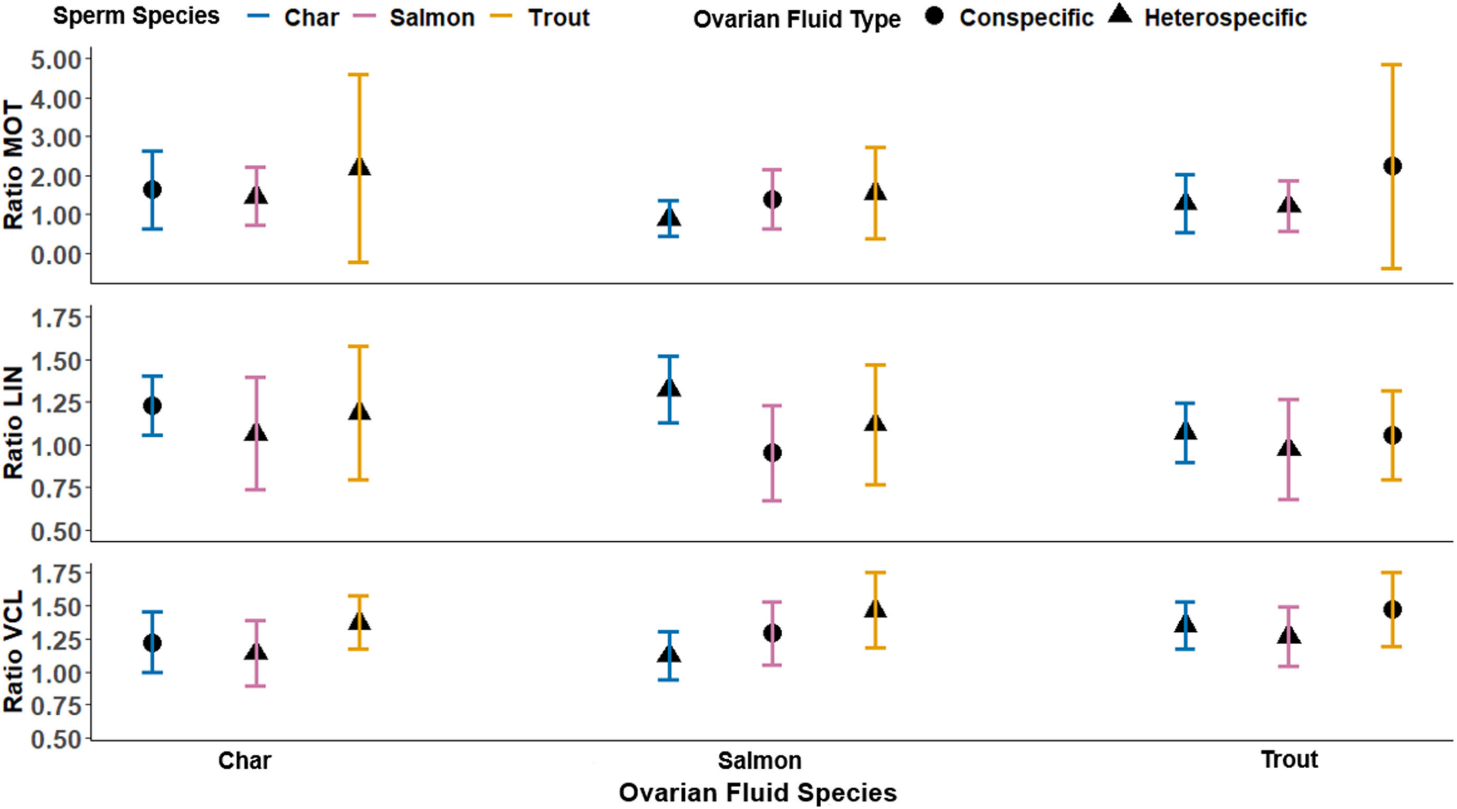
Ratio of (top) sperm motility (% motile) and (middle) swimming linearity (LIN), and (bottom) curvilinear velocity (VCL μm/s) in specific ovarian fluid compared to water from 6.0 to 6.5 s post‐activation – any value above 1.0 indicates ovarian fluid up‐regulated sperm swimming performance. Black shapes represent the average (circles are conspecific sperm to the ovarian fluid, triangles are heterospecific sperm), and colored brackets 2 × standard error among 12 males and females within a species.

Despite individual variation among males in sperm quality, on average sperm of all three species had similar swimming characteristics that declined rapidly post‐activation (Figure S1). A declining function was expected, so we simplified subsequent results and focused on the most biologically relevant time for sperm competition; the earliest we could capture, 6 s. Our first hypothesis, that sperm swimming improves in ovarian fluid when compared to swimming in only water, was in general supported (Figure 2 positive slopes, Figure 3 ratios >1). Individual male performance was visualized as a reaction norm following Purchase et al. (2010) to show this alteration between water and all ovarian fluids combined (Figure 2). Motility (df = 1, *χ* ^2^ = 2.11, *p* = 0.015) and curvilinear velocity (df = 1, *F* = 83.67, *p* < 0.001) were both significantly increased at 6 s on average by 53% (ratio of OF/W = 1.53) and 30% (ratio of OF/W = 1.30), respectively. Linearity was increased on average by 6% (ratio of OF/W = 1.06) but this was not significant (df = 1, *F* = 3.30, *p* = 0.138). Using this approach, we were then able to use the unique ratios of upregulation by each ovarian fluid for each species of sperm to investigate patterns.

Our second hypothesis, that across species, ovarian fluids consistently change sperm swimming to enable conspecific sperm preference was not supported by either of our approaches (Equations 2 and 3). There was variation in how much ovarian fluid modified sperm by male species, but trends were not consistent at 6 s (Figure 3) or throughout the full recorded sperm swimming period (Figure S2). At 6 s post‐activation without differentiating species (Figure 3‐circles vs. triangles), there was no statistically significant difference in how much ovarian fluid modified conspecific versus heterospecific sperm (two heterospecific species averaged—Equation 2) for sperm motility (Figure 3‐top; df = 1, *F* = 1.37, *p* = 0.250), linearity (Figure 3‐middle; df = 1, *F* = 1.60, *p* = 0.210), or velocity (Figure 3‐bottom; df = 1, *F* = 2.36, *p* = 0.133).

For our second approach to hypothesis 2 that differentiated species (higher resolution—Equation 3), and hypothesis 3, that there is a consistent pattern in how ovarian fluid modifies sperm of different heterospecific species, we examined effects at the species level. Reporting general trends (Figure 3), trout ovarian fluid did weakly (see statistics below) change trout sperm more than heterospecific sperm (indicative of conspecific sperm preference), but char and salmon ovarian fluid also tended to change trout sperm more than sperm of their own species (not supporting conspecific sperm preference)—note trout sperm swimming performance in water was not higher than the other species (Figure S1). For motility, the different ovarian fluid species did not alter sperm species differently (interaction not significant; df = 4, *F* = 0.69, *p* = 0.601), indicating no pattern of modification to support hypotheses 2 or 3.

The interaction between male and female species was significant for linearity (df = 4, *F* = 2.78, *p* = 0.034) and velocity (df = 4, *F* = 6.20, *p* = 0.004). The models were thus subsequently broken down and analyzed separately for each ovarian fluid species. There were no differences in modification among the three sperm species by char ovarian fluid (LIN: df = 2, *F* = 0.867, *p* = 0.430; VCL: df = 2, *F* = 3.20, *p* = 0.532) or trout ovarian fluid (LIN: df = 2, *F* = 0.537, *p* = 0.590; VCL: df = 2, *F* = 4.49, *p* = 0.098) – providing no support for hypotheses 2 or 3. There was a difference in sperm modification by salmon ovarian fluid (LIN: df = 2, *F* = 5.23, *p* = 0.011; VCL; df = 2, *F* = 6.17, *p* = 0.005), however not in a manner to support hypotheses (Figure 3). Salmon ovarian fluid changed the LIN of char sperm significantly more than salmon sperm (*p* = 0.008) but there was no difference between char‐trout (*p* = 0.188) and salmon‐trout (*p* = 0.334). For VCL trout sperm were altered significantly more than char sperm (*p* = 0.004), but there was no difference between either trout and salmon (*p* = 0.200) or char and salmon sperm (*p* = 0.200).

## 4 DISCUSSION

Conspecific sperm preference is the last line of defence against hybridization of a female's eggs and has been demonstrated in taxa as diverse as mussels (Klibansky & McCartney, 2014), crickets (Howard et al., 1998; Tyler et al., 2013), birds (Pizzari & Birkhead, 2000; Wagner et al., 2004), and European populations of salmon and trout (Yeates et al., 2013). We therefore expected ovarian fluid mediated modification (documented to enable conspecific sperm preference) of sperm swimming performance would be strong in our hybridizing salmonids. However, while ovarian fluid consistently changed sperm swimming performance when compared to water, it did not do so differently for conspecific than heterospecific sperm, and thus these females cannot bias paternity towards their own species in this way. Given it is the only known mechanism possible for external fertilizing fish, it is therefore possible that cryptic female choice is too weak to promote or maintain reproductive isolation between native North American Atlantic salmon and brook char, nor can it reduce hybridization by invading brown trout.

Ovarian fluid clearly improved sperm motility and velocity. Other studies have shown that components of ovarian fluid (Lehnert et al., 2017; Rosengrave et al., 2009) prolong sperm lifespan and increase sperm velocity in these and related taxa (Elofsson et al., 2006; Evans et al., 2013; Graziano et al., 2023; Purchase & Rooke, 2020; Urbach et al., 2005). Since this function of ovarian fluid was strongly demonstrated, we expected to see conspecific sperm preference. However, ovarian fluid did not upregulate conspecific sperm more than heterospecific sperm in Newfoundland salmonids. Trout ovarian fluid did weakly upregulate trout sperm more, but trout sperm also tended to be upregulated more (not significantly) than the others in the two heterospecific ovarian fluids. This result is surprising because of the high cost to females from fertilization by heterospecific males (Table S1). Atlantic salmon and brook char do not create viable adult hybrids (Chevassus, 1979)—but hybrid mating rates are unquantified and could be high, brown trout and brook char create sterile adults—known as tiger trout (Buss & Wright, 1958), and Atlantic salmon and brown trout create sterile F2s (Chevassus, 1979). In all cases, hybrid fertilizations of a female's eggs create evolutionary dead‐ends. Preventing hybrid fertilizations under heterospecific sperm competition would therefore be highly adaptive.

Yeates et al. (2013) examined hybridization with European populations of Atlantic salmon and brown trout. They found that ovarian fluid was strongly linked to conspecific sperm preference and that the egg itself did not have any protections against hybridization. Ours is the first investigation of conspecific sperm preference for any brook char population. Our study system also has Atlantic salmon that have been isolated from European salmon for 600,000 years (Lehnert et al., 2020), introduced (<150 years) and invasive historically allopatric brown trout, and documented wild hybridization (McGowan & Davidson, 1992). Although as species, salmon and trout evolved together, it is possible allopatric populations of North American salmon may have lost the ability of their European cousins to prevent hybridization by brown trout in sperm competition. Our results suggest cryptic female choice (conspecific sperm preference) via ovarian fluid modification of sperm swimming performance is too weak to prevent hybridization in our study populations. However, to more thoroughly examine cryptic female choice as a means to prevent hybridization in salmonids, more species (including more Genera such as *Oncorhynchus*), and populations over a range of allopatric and sympatric distributions (to determine if this mechanism is only present where historical hybridization pressure exists and hence reinforcement) should be investigated (see examples in Purchase, 2022). Sperm swimming performance evaluations should also be followed by sperm competition experiments to confirm effects on paternity.

## CONFLICT OF INTEREST STATEMENT

The authors declare no conflicts of interest.

## Supporting information


Figure S1

Figure S2

Table S1

Table S2

## Data Availability

The data that support the findings of this study are openly available in Dryad at http://doi.org/10.5061/dryad.wpzgmsbt0.
