# Supplementary figures and images for: Can cryptic female choice prevent invasive hybridization in external fertilizing fish?
Source: Evol Appl. 2023 Jul 13;16(8):1412–21. doi: 10.1111/eva.13573 (PMC10445091; doi:10.1111/eva.13573)

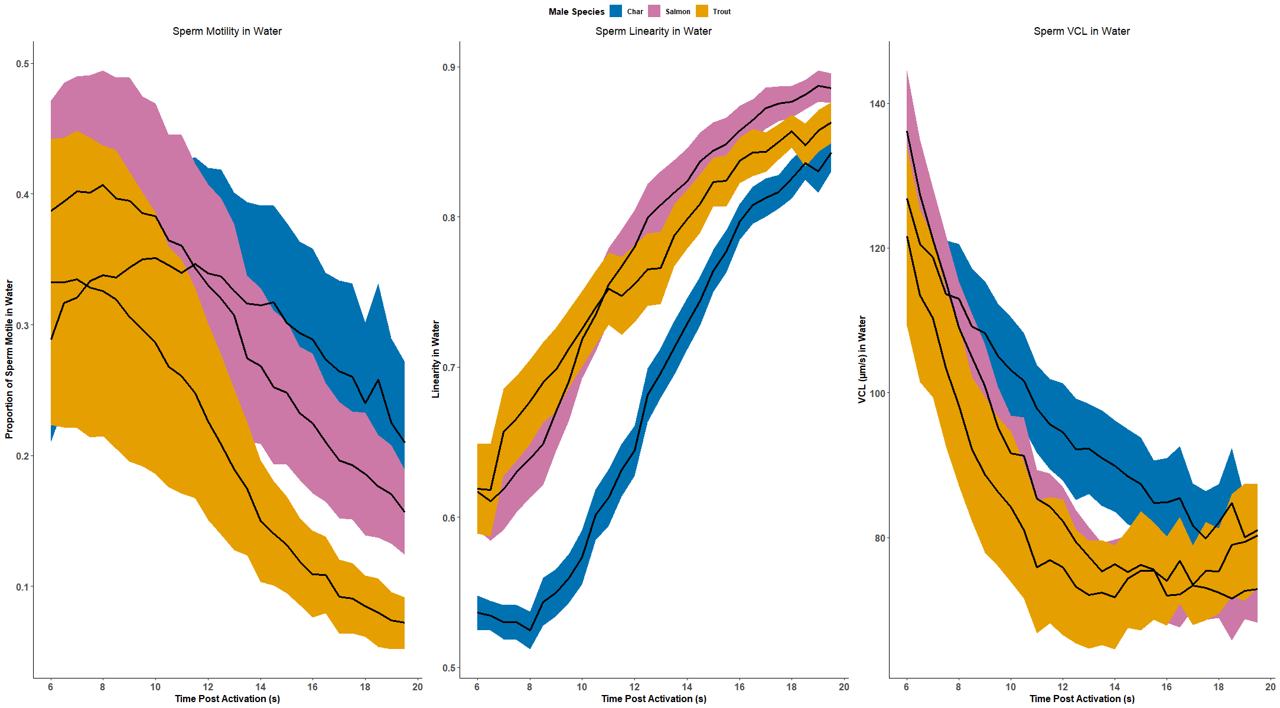

Supplement: Supplementary file 1 — Figure S1 [file EVA-16-1412-s003.png]

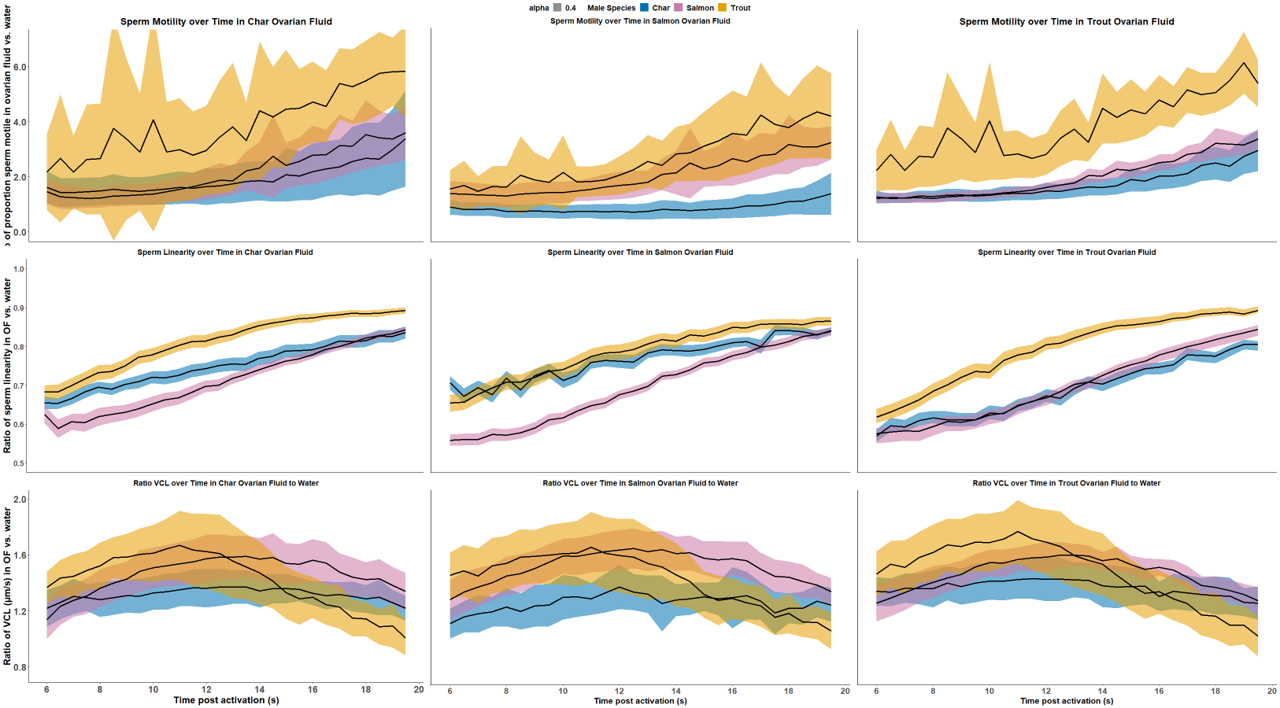

Supplement: Supplementary file 2 — Figure S2 [file EVA-16-1412-s002.png]
