# Supplementary material for: Can cryptic female choice prevent invasive hybridization in external fertilizing fish?
Source: Evol Appl. 2023 Jul 13;16(8):1412–21. doi: 10.1111/eva.13573 (PMC10445091; doi:10.1111/eva.13573)
Supplement: Supplementary file 3 — Table S1 [file EVA-16-1412-s001.docx]

**Supplementary Table 1:** Summary of costs of phylogenetic relatedness, the likelihood of egg exposure, and hybrid fitness. Bold and italicized text indicates the author’s original conclusions. References: 1 (Chevassus, 1979), 2 (Makhrov, 2008), 3 (McGowan and Davidson, 1992b), 4 (Sorenson et al., 1995), 5 (O'Connell, 1982), 6 (Lecaudey et al., 2018), 7 (Sutterlin et al., 1977), 8 (Nygren et al., 1972) 9 (Hartley, 1987), 10 (Buss and Wright, 1958), 11 (Garcia-Vasquez et al., 2004), 12 (Blanc and Chevassus, 1979).

| Hybrid (mother species/ father species) | Salmon/Trout | Trout/Salmon | Trout/Char | Char/Trout | Salmon/Char | Char/Salmon |
| --- | --- | --- | --- | --- | --- | --- |
| Taxonomic relatedness | - **Close** - Same genus | - **Close** - Same genus | - **Most distant** - Different genus - Share less recent common ancestor^6^ | - **Most distant** - Different genus - Share less recent common ancestor^6^ | - **Moderately distant** - Different genus - Share more recent common ancestor^6^ | - **Moderately distant** - Different genus - Share more recent common ancestor^6^ |
| Spawning overlap | - **Very high** - Both species observed to spawn at same place and time^3^ | - **Very high** - Both species observed to spawn at the same place and time^3^ | - **Very high** - Overlap in spawning period and habitat^4^ | - **Very high** - Overlap in spawning period and habitat^4^ | - **Medium, brook char males may still be active during salmon spawning** - Small overlap^3,5^ | - **High, Atlantic salmon sneaker males may be active during brook char spawning** - Small overlap^3,5^ |
| Likelihood of eggs exposed to sperm | - **High** - Both species observed to spawn with each other^3^ | - **High** - Both species observed to spawn with each other^3^ | - **High** - Both species observed to spawn with each other^4^ | - **High** - Both species observed to spawn with each other^4^ | - **Low, brook char males might still be active during salmon spawning.** - Potential brook char sneakers, not known for sure^4^ | - **Medium, brook char females unlikely to be ripe during peak salmon spawning** - **Early Atlantic salmon sneakers may try to fertilize eggs** |
| Fertilization occurs | - Yes^1^ | - Yes^1^ | - Yes^1^ | - Yes^1^ | - Yes^1^ | - Yes^7^ |
| Parent/hybrid chromosome numbers (North American numbers) | - Mother 58^8^ - Father 80^8^ - Hybrid 69^8^ | - Mother 80^8^ - Father 58^8^ - Hybrid 69^8^ | - Mother 80^8^ - Father 84^9^ - No data | - Mother 80^8^ - Father 84^9^ - No data | - Mother 58^8^ - Father 84^9^ - No data | - Mother 84^9^ - Father 58^8^ - No data |
| Offspring hatch | - **Yes, high hatch rate** - 80-100% of the hatch rate of the conspecific cross^1^ | - **Yes, high hatch rate** - 80-100% of the hatch rate of the conspecific cross^1^ | - **Yes, high hatch rate** - 80-100% of the hatch rate of the conspecific cross^1^ | - **Yes, low hatch rate** - <10% of the hatch of the conspecific cross^1^ | - **Yes, medium hatch rate** - 40-80% of the hatch rate of the conspecific cross^1^ | - **Yes, very low hatch rate** - 3% hatch rate in one case^7^ - Total mortality before hatch in another study^12^ |
| Offspring survival to one year | - **F1 high** - 80-100% of the survival of the conspecific cross^1^ - F2 none^2^ | - **F1 high** - 80-100% of the survival of the conspecific cross^1^ - F2 none^2^ | - **F1 medium** - 40-80% of the conspecific cross^1^ | - **F1 low** - <10 percent of the survival of the conspecific cross^1^ | - **F1 low** - <10 percent the survival of the conspecific cross^1^ | - None^1^ |
| Offspring fertility | - F1’s fertile^1^ - Hybrid-hybrid F2’s infertile^2^ - Backcrosses of this cross to salmon produce offspring with high survival and reduced fecundity^11^ | - F1’s fertile^1^ - Hybrid-hybrid F2’s infertile^2^ - Backcrosses of this cross to salmon produce infertile offspring with low survival^11^ | - F1 fertility extremely rare^10^ - Offspring in this case backcrossed with brook trout female resulting in very low survival to fry stage^10^ | - F1 infertile^1^ | - F1 infertile^1^ | - None^1^ |
| Reproductive costs to parents | - **Near total loss by F2 generation** | - **Total loss by F2 generation** | - **Total loss by F1 generation** | - **Total loss by F1 generation** | - **Total loss by F1 generation** | - **Total loss by F1 generation** |
