# Supplementary material for: Can cryptic female choice prevent invasive hybridization in external fertilizing fish?
Source: Evol Appl. 2023 Jul 13;16(8):1412–21. doi: 10.1111/eva.13573 (PMC10445091; doi:10.1111/eva.13573)
Supplement: Supplementary file 4 — Table S2 [file EVA-16-1412-s004.docx]

Supplemental table 2: Casa parameters for sperm swimming characteristic analysis

| **CASA Parameter** | **Value** |
| --- | --- |
| Minimum sperm size (pixels) | 40 |
| Maximum sperm size (pixels) | 440 |
| Minimum track length (frames) | 97 |
| Maximum sperm velocity between frames (pixels) | 8 |
| Minimum VSL for motile (µm/s) | 3 |
| Minimum VAP for motile (µm/s) | 20 |
| Minimum VCL for motile (µm/s) | 45 |
| Low VAP speed (µm/s) | 5 |
| Maximum percentage of path with zero VAP | 1 |
| Maximum percentage of path with low VAP | 25 |
| Low VAP speed 2 (µm/s) | 25 |
| Low VCL speed (µm/s) | 35 |
| High WOB (percent VAP/VCL) | 80 |
| High LIN (percent VSL/VAP) | 80 |
| High WOB two (VAP/VCL) | 50 |
| High LIN two (VSL/VAP) | 60 |
| Frame Rate (frames per second) | 80 |
| Microns per 1000 pixels | 1075 |
